# Development and initial validation of the implicit internalized sexual orientation stigma affect misattribution procedure

**DOI:** 10.3389/fpsyg.2024.1385410

**Published:** 2024-11-29

**Authors:** William J. Hall, Hayden C. Dawes, Jason W. Hannay, Denise Yookong Williams, Joseph J. Frey, Ankur Srivastava, Mimi V. Chapman, Ding-Geng Chen, Amy Blank Wilson, Magdelene E. Ramon, B. Keith Payne

**Affiliations:** ^1^School of Social Work, University of North Carolina, Chapel Hill, NC, United States; ^2^Graduate School of Social Work and Social Research, Bryn Mawr College, Bryn Mawr, PA, United States; ^3^Department of Psychology, University of South Carolina Upstate, Spartanburg, SC, United States; ^4^Department of Social Work, University of North Texas, Denton, TX, United States; ^5^College of Health Solutions, Arizona State University, Phoenix, AZ, United States; ^6^Department of Psychology, University of North Carolina, Chapel Hill, NC, United States

**Keywords:** implicit attitudes, implicit measure, internalized stigma, internalized homophobia, internalized oppression, gay, lesbian, bisexual

## Abstract

**Introduction:**

This article describes the development and initial validation of a measure of implicit internalized stigma among queer people, the Implicit Internalized Sexual Orientation Stigma Affect Misattribution Procedure (Internal-SOS-AMP), a computer-administered sequential priming procedure.

**Methods:**

The creation of the Internal-SOS-AMP involved a mixed-methods approach, including a literature review, expert interviews, stimuli selection and pilot testing, data collection from a large sample, reliability testing, correlational analyses, and confirmatory factor analysis. Psychometric testing was conducted with a national sample of 500 queer adults who completed two waves of data collection. Confirmatory factor analysis was used to evaluate two models: a one-factor model with internalized stigma specified as one overall construct and a two-factor model with internalized stigma specified as two constructs based on binary conceptions of gender (stigma regarding queer women and stigma regarding queer men).

**Results:**

Results showed that the two-factor model best fit the data. This indicates that although implicit attitudes toward queer men and women are highly correlated, implicit internalized stigma differentiated by two gender stimuli groups (men and women) more accurately reflects the data. There was evidence of convergent validity as Internal-SOS-AMP scores showed small positive associations with explicit internalized stigma. Regarding divergent validity, Internal-SOS-AMP scores were inversely related to affirmation of a queer identity. Reliability results for the Internal-SOS-AMP showed good internal consistency and acceptable test–retest reliability.

**Discussion:**

The creation of the Internal-SOS-AMP used best practices for measurement development. Psychometric findings show strong evidence of content validity, convergent validity, divergent validity, and reliability of the Internal-SOS-AMP.

## 1 Introduction

Queer people face substantial mental health disparities, including high rates of depression and suicidality (National Academies of Sciences, Engineering, and Medicine, 2020; Wittgens et al., 2022). The term “queer” is socially constructed and thus is used in different ways to refer to identities, communities, relationships, behaviors, and perspectives regarding sexuality and gender (Hall, 2019; West, 2018). An in-depth discussion of the concept is beyond the scope of this paper. Nonetheless, to clarify, in this paper, we use the term *queer* to refer to various sexual minority (i.e., non-heterosexual) identities, including lesbian, gay, bisexual, pansexual, and queer, as well as the communities based on these identities. In this context, we prefer the term queer over initialisms (e.g., LGB) that may prioritize certain identities and make others invisible. Disparities facing queer people have been linked with various forms of stigma, including internalized stigma (Newcomb and Mustanski, 2010). In the psychological sciences and related fields, internalized stigma has been primarily measured using explicit measures, which typically involve introspection and deliberate, conscious self-reporting on positive and negative statements about being queer using rating scales (Hall et al., 2023). Although attention to implicit measurement of internalized stigma has emerged in recent years, thus far, only one approach to measuring this phenomenon has been proposed (i.e., the Sexuality Implicit Association Test). Because of critiques related to that approach, there is a need for an alternative measure of implicit internalized stigma among queer people that has strong evidence of validity and reliability. In this article, we describe the development and initial validation of such a measure.

### 1.1 Mental health disparities facing queer people

Queer people are a significant minority population who are disproportionately burdened by mental health problems (Institute of Medicine, 2011; National Academies of Sciences, Engineering, and Medicine, 2020). Recent data from the National Survey on Drug Use and Health (NSDUH), a large national U.S. survey, showed substantial mental health disparities by sexual orientation among adults (SAMHSA, 2020). For example, 24–40% of queer people (depending on identity) experienced serious psychological distress (e.g., feelings of anxiety, hopelessness, sadness, and worthlessness) in the past year, compared to 11% of heterosexual people. Beyond distress, 19% of heterosexual people had a diagnosable mental illness as compared to 38–51% of queer people. Some of these mental illnesses were serious enough that they substantially interfered in or limited individuals’ functioning and life activities (5% for heterosexual and 11–22% for queer people). One of the most prevalent mental health problems affecting queer people is depression. In the past year, only 7% of heterosexual people experienced a major depressive episode, yet 16–27% of queer people had a depressive episode (SAMHSA, 2020). Although several mental health disorders are risk factors for suicidality, depression is most often associated with suicide (Tanney, 2000). NSDUH data also showed that compared to their heterosexual counterparts, queer people were significantly more likely to have seriously considered suicide (4% vs. 11–20%, respectively), made plans for suicide (1% vs. 5–8%, respectively), and attempted suicide (0.4% vs. 2–3%, respectively) in the past year (SAMHSA, 2020).

### 1.2 Stigma and minority stress

Leading explanations for the disproportionately high rates of mental health problems among queer people indict social stigma and minority stress as primary causes (Brooks, 1981; Cochran and Mays, 2012; Hatzenbuehler, 2009; Herek, 2016; Herek and Garnets, 2007; Meyer, 2003). *Stigma* refers to a characteristic that marks certain people as different from others, paired with a shared social understanding that the targets of social stigma are inferior or of less value, leading to prejudice, stereotyping, and discrimination (Herek, 2015). In U.S. society, queer people have been and continue to be stigmatized and disproportionately exposed to social stressors that derive from stigmatization which can compromise their mental health (Brooks, 1981; Cochran and Mays, 2012; Hatzenbuehler, 2009; Herek, 2016; Herek and Garnets, 2007; Meyer, 2003; National Academies of Sciences, Engineering, and Medicine, 2020). *Minority stress theory* posits that queer people experience not only typical life stressors (e.g., death of a loved one, personal injury, financial hardship), but also an additional layer of stressors related to their stigmatized minority identity (Brooks, 1981; Meyer, 2003). These additional stressors include prejudice events (e.g., violence, discrimination, and rejection) and proximal stressors, including expectations of prejudice events, pressure to conceal one’s sexual identity, and internalized stigma. These stressors—specific to queer people’s minoritized sexual orientation—can be burdensome and harmful, often leading to mental health disparities.

### 1.3 Internalized stigma

One of the most prominent stressors in minority stress theory (Meyer, 2003) is *internalized stigma*, which occurs when members of a minority group internalize negative social views about their identity (David and Derthick, 2014). Various terms have been used to refer to internalized stigma among queer people, such as internalized homophobia, internalized biphobia, internalized homonegativity, internalized heterosexism, internalized oppression, and sexual self-stigma (Herek et al., 2015). No matter the name, internalized stigma refers to queer people mentally incorporating negative attitudes and beliefs pervasive in homophobic and heterosexist social systems (Szymanski et al., 2008a). In U.S. society, queer people grow up and live in a culture in which heterosexuality is the norm, ideal, and expectation. Americans are inundated with sociocultural messages that value heterosexuality and stigmatize queer identities, permeating schools, homes, workplaces, faith communities, and the media (Hall, 2019). Thus, through socialization processes, queer people inadvertently learn and incorporate negative views about their identity. Some negative self-attitudes queer people hold include believing that their desires, identities, and relationships are abnormal, immoral, or psychopathological. In turn, these negative self-views are associated with negative affect (e.g., feelings of shame, worthlessness, and guilt). An empirical literature review found significant associations between internalized stigma and low self-esteem, as well as elevated rates of depression, suicidal ideation and behavior, non-suicidal self-injurious behavior, anxiety, and negative affect (e.g., feeling demoralized, lonely, distrustful, guilty, and ashamed; Szymanski et al., 2008b). Similarly, meta-analytic findings evidenced a significant moderate effect (*r* = 0.26) for the relationship between internalized queer stigma and mental health outcomes (i.e., symptoms of depression and anxiety; Newcomb and Mustanski, 2010).

Another important feature of internalized stigma is that it can be *explicit* or *implicit*. *Explicit attitudes* involve thoughts and feelings of which individuals are consciously aware and can easily report on, whereas *implicit attitudes* are automatically activated and conceivably exist outside of a person’s conscious awareness, making implicit attitudes difficult to access, monitor, and control (Carlston, 2010; Hall et al., 2023). Although some queer individuals might be consciously aware of stigma they may have internalized, many are likely unaware of deeply held attitudes and beliefs existing on the margins of awareness—known as *implicit internalized stigma* (Hall et al., 2023). Over the last 50 years, there has been a drive in the queer community toward pride—feeling proud of being queer in the face of an oppressive social world (Halperin and Traub, 2009). A vast majority of queer people would undoubtedly consciously repudiate statements, such as “being queer is abnormal” or “queer people are sick,” yet such negative self-attitudes and self-beliefs may be repressed and hidden to some individuals as implicit internalized stigma, which can influence queer people’s emotional, cognitive, and behavioral functioning and wellbeing (Halperin and Traub, 2009; Hatzenbuehler et al., 2009; Nadal and Mendoza, 2014). Negative implicit attitudes toward queer people are much more prevalent in the U.S. population than negative explicit attitudes (68% vs. 42%, respectively; Nosek et al., 2007; Saad, 2012); thus, implicit internalized stigma may likely have a more widespread, insidious effect on mental health among queer people.

### 1.4 Measurement of internalized stigma

Research assessing internalized stigma among queer people has relied almost exclusively on explicit measurement (Berg et al., 2016; Grey et al., 2013; Newcomb and Mustanski, 2010; Szymanski et al., 2008b; Williams et al., 2023), which has typically involved introspective self-report questionnaires on which respondents rate their agreement/disagreement with a series of statements about being queer. These explicit measures may not be able to capture deeply encoded attitudes that can be automatically activated even if they are not intentionally endorsed. And, the validity of these measures may be compromised by social desirability bias whereby participants likely respond in more positive ways to statements about queer people (e.g., “Homosexuality is a sexual perversion.”; Rudman, 2011). Thus, investigators interested in measuring implicit attitudes should use indirect measures to infer respondents’ attitudes based on their behavior or performance on a task.

#### 1.4.1 Implicit Association Test

Although numerous measures of *explicit* internalized queer stigma are available, only one recognized measure of *implicit* internalized queer stigma is currently available: the Sexuality version of the Implicit Association Test (IAT; Nosek et al., 2007). Although the IAT was a pioneering measure in implicit attitude scholarship, research indicates several limitations in reliability and validity for the Sexuality IAT. The IAT asks respondents to rapidly categorize 2 target concepts (e.g., heterosexual vs. gay) using 2 sets of opposing attributes (e.g., good vs. bad); respondents’ reaction times are measured and compared to determine the strength of associations. Faster associations are interpreted as stronger implicit attitudes. However, the complex dual-task structure of the IAT leads respondents to use various strategies to simplify and ease the burden of responding. As such, instead of measuring a person’s implicit mental associations, the IAT may be measuring task-switching ability, perceived similarity, attentional asymmetries, or executive function (Karpinski and Hilton, 2001; Klauer and Mierke, 2005; Olson and Fazio, 2004; Rothermund and Wentura, 2004), which calls the construct validity of the IAT into question.

The IAT may also lack reliability. Of the studies conducted using the Sexuality IAT with queer participants (Anselmi et al., 2015; Bankoff et al., 2015; Chen and Chang, 2020; Dhabhar and Deshmukh, 2021; Fleming and Burns, 2016; Hatzenbuehler et al., 2009; Jellison et al., 2004; Jones and Devos, 2014; Millar et al., 2016), only one study reported internal consistency reliability and found low reliability (*α* = 0.54; Millar et al., 2016). No studies were found of the test–retest reliability of the Sexuality IAT with queer samples. However, based on 0.70 as the common cutoff for acceptable reliability, a number of studies have shown that other versions of the IAT had unacceptably low or unstable test–retest reliability (*r* = 0.25 to 0.69; Lane et al., 2007; Rezaei, 2011). Moreover, the test–retest reliabilities did not depend on the length of time; poor reliability values were similar whether the retest was completed within the same session or after 1 year (Egloff et al., 2005).

Another issue with the Sexuality IAT is that some of its stimuli are outdated and only tangentially related to the construct of interest. For example, the Sexuality IAT uses the term “homosexual,” which has become outdated and pejorative; the terms “gay” or “lesbian” have supplanted “homosexual” (GLAAD, 2024). In addition to verbal stimuli, the Sexuality IAT uses images, including figurines of couples that top wedding cakes and figures often found on bathroom signs paired together to represent heterosexual couples, same-sex male couples, and same-sex female couples. The Sexuality IAT was intended to measure attitudes about heterosexual and queer people; however, none of the stimuli images depict real people. Prior implicit research found that target stimuli of pictures of individuals was significantly correlated with an approach/avoidance task regarding social groups; however, when target stimuli of words were used, no associations were found with approach/avoidance scores (Foroni and Schubert, 2007). It may be that pictures of actual people activate socio-emotional responses more so than more abstract stimuli, such as words or symbols (Foroni and Bel-Bahar, 2010). Further, images of wedding cake figurines and symbols for men and women on bathroom signs might conflate attitudes about queer people with attitudes about civil rights facing the queer community, including same-sex marriage or bathroom bills requiring people to use the bathroom corresponding to their sex assigned at birth, not their gender identity. In addition, the attributes used in the IAT (e.g., *pleasure*, *marvelous*, *wonderful*, *beautiful*, *terrible*, *awful*, *joyful*, *humiliate*, *horrible*, *agony*, *glorious*, *lovely*, *painful*, *nasty*, and *tragic*) are broad, general attributes that often do not represent specific attitudes of internalized stigma. Negative attitudes regarding queer people fall under several content domains: deviance/perversion, unnaturalness, abnormality/pathology, hypersexuality, and immorality (Hall, 2019; Nadal and Mendoza, 2014; Szymanski et al., 2008a; Szymanski et al., 2008b; Williamson, 2000).

#### 1.4.2 Affect Misattribution Procedure

The Affect Misattribution Procedure (AMP) is a computer-administered sequential priming procedure that measures implicit attitudes toward social groups (Payne et al., 2005). In the AMP, participants are first presented with a prime (e.g., a picture of a heterosexual couple or same-gender couple), followed by an affectively neutral image (e.g., a Chinese pictograph). Participants are then asked to judge the meaning of the Chinese pictograph using positive and negative response options (e.g., “pleasant” or “unpleasant”). Participants’ responses are influenced by the primes because participants appraise the neutral stimulus (Chinese pictograph) more positively when the prime image presented is inherently positive to the participant rather than negative, which indicates that affective reactions to the primes are being mistakenly attributed to the neutral stimuli.

The AMP has been well tested and has substantial evidence of reliability and validity as a measure of implicit attitudes (Payne and Lundberg, 2014). Meta-analytic findings show that the average internal consistency reliability of the AMP is high (*α* = 0.81; Payne and Lundberg, 2014). Another meta-analysis examining the AMP’s predictive validity found a moderately strong mean effect size between AMP scores and human behavior (*r* = 0.35; Cameron et al., 2012). This meta-analysis also found evidence of convergent validity, with an overall significant medium effect size for the relationship between AMP scores and explicit attitude measures (*r* = 0.30; Cameron et al., 2012). The simplicity of the design is one of the distinct advantages of the AMP because approximately 50 trials of the AMP can be completed in about 1 min (Payne and Lundberg, 2014). In addition, participants are half as likely to stop participating during the AMP as opposed to the IAT (DeBell et al., 2010). Many versions of the AMP have been developed and used in research to measure attitudes related to race, ethnicity, gender, age, politics, and substance use (Payne and Lundberg, 2014).

### 1.5 Rationale for and goals of the current study

Given that research and clinical practice have almost exclusively focused on explicit internalized stigma and that the only measure of implicit internalized stigma (the Sexuality IAT) has limited and questionable validity and reliability, there is a need to develop a new measure of implicit internalized stigma for queer people. Such a measure could be useful in descriptive and etiological research on the internalization of implicit stigma, predictive studies on implicit stigma and mental health disparities, and intervention research to mitigate implicit internalized stigma and its role in health disparities.

The purpose of this study was to develop a new measure of implicit internalized stigma among queer people and examine its psychometric properties. This involved the creation of a version of the AMP, referred to as the *Implicit Internalized Sexual Orientation Stigma AMP (Internal-SOS-AMP)*. Psychometric testing focused on content validity, convergent validity, divergent validity, and reliability. The study was driven by the following research questions: (1) Does the Internal-SOS-AMP measure specific primary dimensions of implicit internalized stigma among queer people? (2) To what extent does evidence support the interrelationships between these dimensions? (3) How are scores on the Internal-SOS-AMP related with scores from measures of constructs theoretically and empirically related and unrelated to implicit internalized stigma? (4) Does the Internal-SOS-AMP have evidence of acceptable internal consistency reliability and test–retest reliability? For the second research question, we hypothesized that implicit internalized stigma could be modeled two ways with acceptable fit: (a) As a one-factor model with all of the Internal-SOS-AMP scores as indicators of one latent construct (i.e., implicit internalized queer stigma), and (b) as a two-factor model with internalized stigma as two latent constructs based on two gender stimuli groups (i.e., stigma regarding queer women and stigma regarding queer men). For the third research question, we hypothesized that small positive correlations would be found between implicit internalized stigma and explicit internalized stigma, based on prior theory and research (e.g., Cameron et al., 2012; Hofmann et al., 2005; Nosek and Smyth, 2007), and medium inverse correlations would be found between implicit internalized stigma and affirmation of queer identity, based on prior theory and research (e.g., Paul et al., 2014; Toomey et al., 2016).

## 2 Methods

The creation of the Internal-SOS-AMP involved a mixed-methods approach: literature review, expert interviews, selection and pilot testing of stimuli, and data collection from a large sample for psychometric testing. This study was approved by the authors’ institutional review board.

### 2.1 Literature review: conceptual, empirical, and measurement literature

As a precursor to creating the Internal-SOS-AMP, the first author reviewed empirical and theoretical/conceptual literature on the construct of internalized stigma among queer people. Papers reviewed included Berg et al. (2016), Herek (2000, 2007), Herek et al. (2009), Nadal and Mendoza (2014), and Szymanski et al. (2008a, 2008b). In addition, the author reviewed the most widely used measures of explicit internalized stigma: Nungesser Homosexuality Attitudes Inventory (Nungesser, 1983; Radonsky and Borders, 1996; Shildo, 1994), Internalized Homophobia Scale (Herek et al., 1998; Martin and Dean, 1987), Internalized Homophobia Scale (Wagner et al., 1994; Wagner et al., 1996), Internalized Homophobia Scale (Ross and Rosser, 1996), Internalized Homonegativity Inventory (Mayfield, 2001), and the Lesbian Internalized Homophobia Scale (Szymanski and Chung, 2001); as well as the only measure of implicit internalized queer stigma (i.e., the Sexuality IAT; Banse et al., 2001; Steffens, 2005). This review of the scholarly literature and extant measures provided insight into the conceptualization and operationalization of internalized stigma. Multiple dimensions of internalized stigma were found, including domains around deviance and social unacceptability; unnaturalness or not biologically correct; abnormality, psychopathology, and illness; immorality and sinfulness; negative feelings (e.g., sadness and discomfort); negative stereotypes (e.g., sexual predators, hypersexuality); and shame-related behaviors (e.g., seeking to change one’s sexual orientation, avoidance of queer people). Extant theory and research show positive associations between these constructs (e.g., Herek, 2000; Nadal and Mendoza, 2014; Szymanski et al., 2008a, 2008b). The measures review also revealed that the initial measures of internalized stigma were developed based on and for gay men (i.e., Martin and Dean, 1987; Mayfield, 2001; Nungesser, 1983; Wagner et al., 1994), with few measures for gay/lesbian women and other identities (e.g., bisexual). On one hand, this reflects a partiality in research that has historically prioritized cisgender gay men over others in the queer community (e.g., Berg et al., 2016); and on the other hand, this acknowledges that although there are common aspects of internalized stigma across queer identities (e.g., shame and discomfort), there are also aspects unique to certain identities, such as internalized stereotypes for gay men and gay/lesbian women.

### 2.2 Expert interviews

In addition to the literature review, we conducted interviews with 5 experts with clinical, conceptual, and/or empirical expertise on sexual minority stigma to identify dimensions of internalized stigma that should be reflected in the Internal-SOS-AMP response options. Experts were identified by searching the literature for publications as first author in the past 15 years on the topic of internalized stigma among queer people. Two study team members reviewed the curriculum vitae of identified experts to gauge the extent and depth of work with the topic and population, and then those with higher experience (i.e., number of publications and presentations on the topic, years of practice experience with the population and topic) were selected by the first author and invited to be interviewed. The interviews were unstructured and open-ended discussions beginning with the main areas of queer internalized stigma, followed by probes to identify examples of specific word pairs that represented stigma areas discussed. Interviews lasted approximately 45 to 60 min and were conducted by the first author.

Interview transcripts were analyzed by the first two authors to identify dominant themes concerning the dimensions of internalized stigma. In terms of positionality, these researchers both identified as queer, cisgender men; one is White and one is Black in terms of race/ethnicity. Their upbringing ranged from moderate to conservative sociopolitical backgrounds. Both are from Christian religious backgrounds. And, both have a social work and mental health background, and both have had their own personal struggles with internalized stigma. These backgrounds and experiences may have influenced the identification of themes from the interview data, including honing in on themes around abnormality, immorality, negative affect, and negative stereotypes of queer men. However, strategies for rigor were used during coding to bolster trustworthiness, including having multiple researchers coding and analyzing the data as well as keeping an audit/decision trail to document steps in the data collection, coding, and analysis (Padgett, 2012).

Interview data were analyzed using qualitative content analysis with a conventional approach, which allows themes to emerge from the data (Hsieh and Shannon, 2005). First, the two authors independently read, wrote memos, and open-coded qualitative responses. Second, the pair met and compared notes, discussed codes, and derived a final coding scheme. Third, the authors independently reread the interview responses and coded text data using the established coding scheme. Finally, the pair met to compare the results of their coding and resolved a few discrepancies through negotiated consensus. Five thematic categories emerged from the interview data, and within each of these categories were numerous potential response option pairs: (1) morality/immorality (e.g., moral/immoral, good/bad, right/wrong), (2) abnormality/deviance (e.g., normal/abnormal, healthy/sick, natural/unnatural), (3) positive and negative affect (e.g., pleasant/unpleasant, appealing/unappealing, comfortable/uncomfortable), (4) stereotypes (e.g., stable/unstable, gentle/predatory, wholesome/promiscuous), and (5) a miscellaneous category (e.g., lovable/unlovable, fortunate/unfortunate, genetic/choice).

### 2.3 Selection of response domains

The themes from the expert interviews and domains identified in the literature review provided triangulation in understanding the phenomenon of internalized stigma. There was overlap between the thematic categories discussed by experts and the ways internalized stigma had been conceptualized and measured as evident from the review of the literature and extant measures (see Table 1). Four members of the study team met and discussed which domains and associated response options should be included in the Internal-SOS-AMP. Given the study measurement goal to identify coherent and salient response option pairs for internalized stigma, the stereotypes and miscellaneous categories were eliminated because many of the stereotypes were specific to subgroups of the queer community (e.g., “gay men are sexual predators” and “bisexual people are promiscuous”) rather than the community generally and the miscellaneous category did not represent a coherent conceptual dimension. Shame-related behaviors were not able to be used because the AMP captures affective and cognitive representations, not self-reports of individual behavior. Conceptual overlap was present in the abnormality/deviance theme and the domains of deviance, perversion, abnormality, and unnaturalness. Alignment was found regarding immorality and negative affect/feelings. Response option pairs from the expert interviews were ranked by the first two authors based on how well they represented the three dominant categories of internalized stigma (i.e., abnormality/deviance, immorality, negative affect/feelings). Ultimately, four pairs of response options were chosen (i.e., moral/immoral, normal/abnormal, appealing/unappealing, and pleasant/unpleasant) to be included in the Internal-SOS-AMP, which represented the three dominant dimensions of internalized stigma. Four pairs were chosen so that the measure would not be burdensome for participants. To balance the response options, two pairs were chosen from the positive and negative affect category, reflecting general affect, and two pairs were chosen from the more specific categories of morality/immorality and abnormality/deviance.

**Table 1:** Domains of internalized stigma identified from literature review and expert interviews.

Domains from literature review	Themes from expert interviews
Deviance Perversion Socially unacceptable	Abnormality/Deviance
Abnormal Pathology or Psychopathology Illness or Mental illness Disordered	
Unnatural Not biologically correct	
Immoral Sinful Religiously unacceptable	Immorality
Negative feelings (e.g., sadness, discomfort, shame, isolation)	Negative affect (e.g., unpleasant, unappealing, uncomfortable)
Negative stereotypes (e.g., sexual predators, hypersexuality)	Negative stereotypes (e.g., unstable, predatory, promiscuous)
Shame-related behaviors	Miscellaneous (e.g., unlovable, unfortunate)

### 2.4 Selection and pilot testing of stimuli

A pool of prime images of couples was gathered from a leading stock photography company after preliminary searches of several company websites. Images of couples were sought because evidence shows that images containing more than one person lead to better elicitation of implicit attitudes compared to images of individuals, (Cooley and Payne, 2017; Cooley and Payne, 2019). Initial image searches revealed that there were very few images of older queer couples; therefore, we focused on images of couples who appeared to be age 25–40. In addition, there were fewer images of queer couples than heterosexual couples and fewer images of queer couples of color than queer White couples. Images of inter-racial queer couples were rare. Also, almost all of the couple images depicted seemingly cisgender men or women; there were almost no images of gender diverse individuals (e.g., transgender, genderqueer, and non-binary people) depicted in intimate dyads. An initial pool of 342 images of couples was compiled and then narrowed to 118 images by the first author. Images of couples in non-intimate poses that may have been viewed as friends were cut. In addition, heterosexual images were cut if couples looked outside the age band, were inter-racial couples, or were extremely attractive (i.e., obvious models as opposed to everyday people). Data were needed to further narrow the 118 images and balance them across the sexual orientation groups. These data were collected from a group of 100 task workers registered with Mechanical Turk, an online crowdsourcing service; tasks needing completion are posted on the Mechanical Turk website, workers select and complete the tasks, and then they are paid. We asked workers to view the 118 images and rate each image on *romantic involvement* (“Do you think these two people are romantically involved with each other?”), *attractiveness* (“How attractive is this couple?”), *intimacy* (“How intimate is this couple?”), *closeness* (“How close are these two people with each other?”), *naturalness* (“How natural does this picture look?”), and *happiness* (“How happy is the couple in this picture?”). The response options for the romantic involvement question were *Yes* or *No*. The response options for attractiveness, intimacy, closeness, naturalness, and happiness were on a 10-point scale (*Not at all* – *Extremely*). Mean scores for each of the 118 images, based on these six variables were used to eliminate images in order to find a balanced set of images of couples across the three sexual orientation groups (heterosexual couples, queer men couples, and queer women couples) where mean scores for each group would be similar. Balance was achieved for all of the variables except for attractiveness where queer men couples were rated slightly lower (*M* = 6.9) than queer women couples (*M* = 7.4) and heterosexual couples (*M* = 7.5), as well as naturalness where heterosexual couples were rated slightly higher (*M* = 7.8) than queer women couples (*M* = 7.2) and queer men couples (*M* = 7.0). The final image set (*N* = 30) included 10 images per sexual orientation group, with racial/ethnic diversity in each group (one Asian couple, two Black couples, two Latine couples, and five White couples). Couple images were purposefully balanced to include five White couples and five couples of color to negate potential effects of internalized racist attitudes; as described later, implicit internalized stigma scores are aggregated.

The stimuli selected for the affectively neutral images were Tibetan words because very few Americans can read Tibetan (Samten and Sharngoe, 2020). A translation website was used to translate common words in English (e.g., “again”) to Tibetan words (e.g., “འངའིན”) and then images of those words were used as stimuli.

### 2.5 Data collection for psychometric testing

#### 2.5.1 Procedure

A sample of queer adults was recruited through CloudResearch panels (formerly TurkPrime), a participant-sourcing platform for online research (Litman et al., 2017). Given that the queer community is a hard-to-reach population (Ellard-Gray et al., 2015; Meyer and Wilson, 2009), the substantial pool of potential participants available through CloudResearch was used to create a panel. Research shows that CloudResearch participants are diverse, have demographic characteristics that approximate nationally representative U.S. samples, and are more representative than in-person or Internet-based convenience samples (e.g., Buhrmester et al., 2016; Chandler et al., 2019; Coppock, 2019; Krupnikov and Levine, 2014; Levay et al., 2016; Mortensen and Hughes, 2018; Mullinix et al., 2015; Shapiro et al., 2013).

An online survey was administered at two time points to the same sample. The Wave 1 survey was launched in early November 2020 and participants responded to demographic questions, followed by stressor items, the Internal-SOS-AMP, and then outcome questions. The Wave 2 survey was administered at the end of January 2021 and included most of the same items as the Wave 1 survey. Each participant was paid $10 for completing the surveys, which is standard for CloudResearch participants. Two participation validity check items were embedded in the survey (e.g., “Please select the ‘strongly agree’ response for this item”) to identify participants who did not read the survey items and may have responded aimlessly. Any participant who failed one of these items was excluded (*n* = 31). In addition, participants who only completed a small part of the initial survey items and then stopped participating were excluded (*n* = 81).

#### 2.5.2 Participants

The final sample included 500 participants who completed the Wave 1 survey, and 358 of these respondents completed the Wave 2 survey (return rate = 71.6%). Ages of participants ranged from 18 to 70 years, with an average age of 33.7 (*SD* = 10.0). In terms of race/ethnicity, 64.8% of participants were White, 13.0% were Black or African American, 11.0% were Hispanic/Latine, 6.2% were Asian or Pacific Islander, 2.8% were Native American, and 2.2% were multiracial. For birth sex, 60.2% of participants were assigned female and 39.8% were assigned male. In terms of gender identity, 86.0% of participants were cisgender and 14.0% reported a transgender identity (e.g., transgender man, transgender woman, genderqueer). For sexual orientation, 61.0% of participants identified as bisexual, pansexual, or bisexual or pansexual and another identity; 29.8% identified as gay or lesbian; 8.4% identified as queer; and 0.8% indicated another identity (e.g., demisexual). A large majority of participants (84.0%) indicated that they did not have a disability and 16.0% had a disability. Similarly, 85.4% of participants were U.S.-born, and 14.6% were first-or second-generation immigrants. For highest education level achieved, 0.6% of participants had less than a high school degree, 8.2% were high school graduates, 25.2% had some college but no degree, 11.6% had an associate degree, 35.0% had a bachelor’s degree, 17.0% had a master’s or professional degree, and 2.4% had a doctoral degree. In terms of self-reported income level, 22.2% of participants were low income, 29.4% were lower-middle income, 39.4% were middle income, 9.0% were upper-middle income, and 0.2% were high income. Participants’ current geographic region varied: 32.9% were in the South, 21.6% were in the Midwest, 20.8% were in the Northeast, 20.2% were in the Pacific-West region, and 4.4% were in the Mountain-Plains region.

#### 2.5.3 Variables and measures

##### 2.5.3.1 Implicit internalized stigma

**Figure 1:**
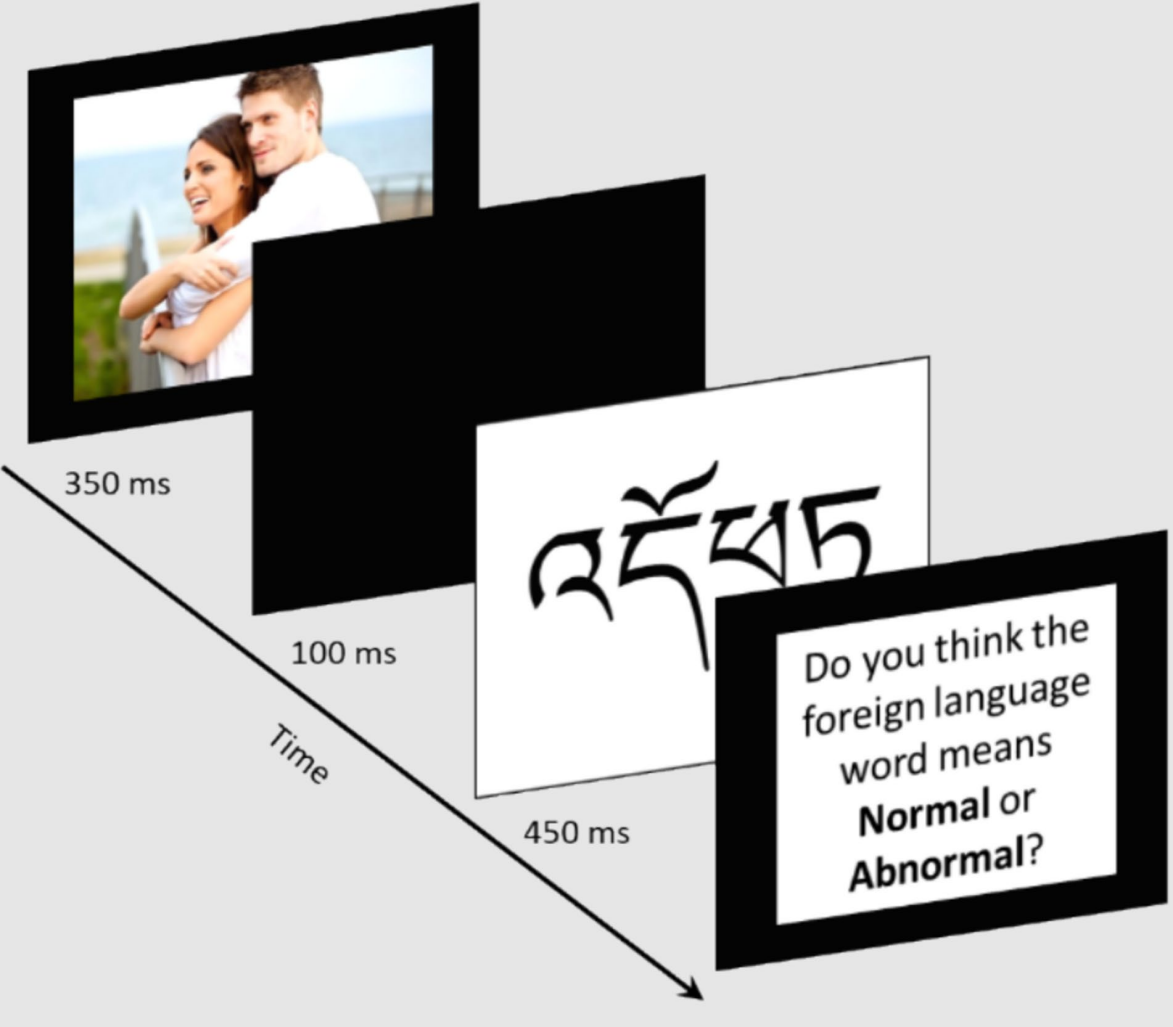
Sample trial of the internal-SOS-AMP. Couple images were purchased with no attribution required.

In the Internal-SOS-AMP, participants were first presented with a prime (i.e., a picture of a heterosexual couple, queer women couple, or queer men couple) for 350 ms, followed by a blank screen for 100 ms, a Tibetan word for 450 ms, and finally a black screen that remained until participants responded to the question if they thought the Tibetan word meant *pleasant* or *unpleasant*, *normal* or *abnormal*, *appealing* or *unappealing*, or *moral* or *immoral* (Figure 1). The Internal-SOS-AMP consisted of 120 trials: 40 trials for each sexual orientation group (heterosexual couples, queer women couples, and queer men couples), with 10 trials for each response option pair (moral/immoral, normal/abnormal, appealing/unappealing, and pleasant/unpleasant). Positive responses (i.e., moral, normal, appealing, pleasant) were coded as 1, and negative responses (i.e., immoral, abnormal, unappealing, unpleasant) were coded as 0. Internal-SOS-AMP composite scores were computed by subtracting the proportion of positive responses following queer couple primes from the proportion of positive responses following heterosexual couple primes. Therefore, a composite score of 0 is completely neutral, indicating no implicit internalized stigma, scores >0 indicate some level of negative attitudes toward queer people (i.e., implicit internalized stigma), and scores <0 indicate positive implicit attitudes toward queer people.

##### 2.5.3.2 Explicit internalized stigma

Explicit internalized stigma was measured using the Internalized Homonegativity Inventory (IHNI; Mayfield, 2001). Participants rated their level of agreement on 11 items focused on *personal homonegativity* (e.g., “I feel ashamed of my sexuality”) and 5 items on the *morality of homosexuality* (e.g., “I believe it is morally wrong for people to be attracted to the same sex”) using a 7-point Likert response scale ranging from *Strongly Disagree* (1) to *Strongly Agree* (7). An overall explicit internalized stigma score was attained by averaging responses on the 16 items, with higher scores indicating higher levels of explicit internalized stigma. The internal consistency reliability of this measure was very good (*α* = 0.94).

##### 2.5.3.3 Affirmation of queer identity

Affirmative attitudes about one’s queer identity were measured using the *gay affirmation* subscale of the IHNI (Mayfield, 2001). Participants rated their level of agreement on 7 items (e.g., “I am thankful for my sexual orientation”) using a 7-point Likert response scale ranging from *Strongly Disagree* (1) to *Strongly Agree* (7). An affirmation of queer identity score was calculated by averaging responses, with higher scores indicating more positive attitudes about one’s queer identity. The internal consistency reliability of this measure was very good (*α* = 0.84).

#### 2.5.4 Data analysis

**Figure 2:**
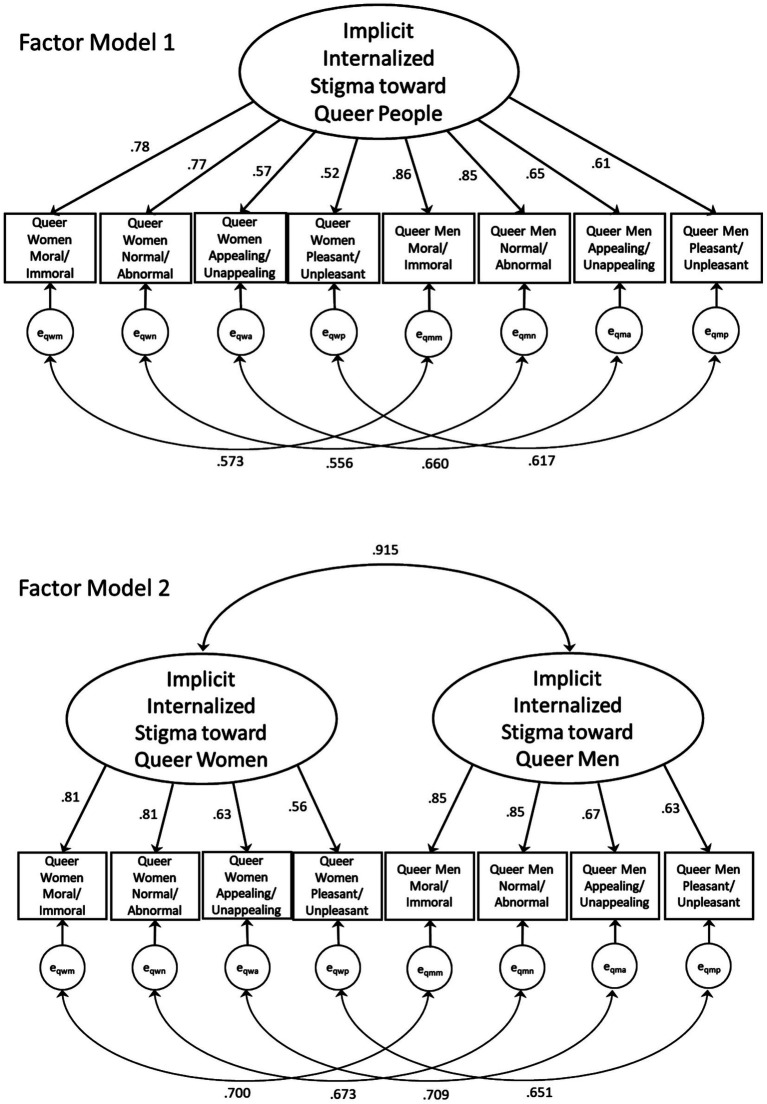
Internal-SOS-AMP confirmatory factor analysis models (*N* = 500). Latent constructs are shown as ellipses, indicator variables are shown as rectangles, and error terms are shown as circles.

Analyses were performed to examine the validity and reliability of the Internal-SOS-AMP. First, descriptive statistics were run to assess the central tendency and dispersion of scores. Second, confirmatory factor analysis (CFA) was used to evaluate the factor structure of the Internal-SOS-AMP. Following best practices in CFA (Bowen and Guo, 2012), multiple competing measurement models were specified and examined: a one-factor model with eight indicators and a two-factor model with four indicators per factor (Figure 2). In the one-factor model, all of the Internal-SOS-AMP scores are indicators of one underlying latent construct: implicit internalized queer stigma. In the two-factor model, internalized stigma is specified as two latent constructs based on two gender stimuli groups: stigma regarding queer women and stigma regarding queer men. Both of these specified models can be supported by prior research and theory because implicit stigma has been conceptualized and studied among the queer community broadly (e.g., Mayfield, 2001) and for dominant subgroups, including sexual minority men and women (e.g., Szymanski and Chung, 2001; Theodore et al., 2013). Therefore, we hypothesized that both the one-factor and two-factor models would have adequate fit. Mplus (version 8) was used to perform the CFA. The indicator variables were continuous and approximately normally distributed (i.e., skewness ranging from −0.01 to 0.69 and kurtosis ranging from 0.90 to 2.21) based on findings from Curran et al. (1996). Thus, maximum likelihood estimation was used (Muthén and Muthén, 2017). The quality of each model was evaluated using multiple fit criteria: comparative fit index (CFI) ≥ 0.95, Tucker-Lewis index (TLI) ≥ 0.95, root mean square error of approximation (RMSEA) ≤ 0.06, and standardized root mean square residual (SRMR) < 0.08, recommended by Hu and Bentler (1999) and West et al. (2012). None of these variables were missing values. Third, bivariate Pearson correlations were used to examine the extent to which scores on one indicator were related to scores on all other indicators. These results can indicate the extent to which indicators are assessing the same or similar content and may reveal indicator redundancy (Swerdlik and Cohen, 2005). Fourth, to assess divergent validity, Pearson correlations were run between Internal-SOS-AMP composite scores and the composite score for explicit internalized stigma. To assess convergent validity, Pearson correlations were run between Internal-SOS-AMP composite scores and the affirmation of queer identity composite score. Fifth, reliability analyses were performed to examine correspondence and consistency in the Internal-SOS-AMP. To assess internal consistency reliabilities, Cronbach’s alphas were computed for each Internal-SOS-AMP score. To assess test–retest reliability, intraclass correlation coefficients (ICCs) were computed using two-way mixed-effects models for absolute agreement and average measures because the same group of participants completed the same measure at two time points and we were interested in the extent to which participant scores were the same across time points (McGraw and Wong, 1996; Shrout and Fleiss, 1979).

## 3 Results

The results of psychometric testing of the Internal-SOS-AMP included several pieces of evidence: descriptive statistics of Internal-SOS-AMP scores; confirmatory factor analysis results; correlations between Internal-SOS-AMP scores; correlations between Internal-SOS-AMP scores and two scales of constructs theoretically related and inversely related to internalized implicit stigma (i.e., explicit internalized stigma and affirmation of queer identity, respectively); and internal consistency reliability and test–retest reliability.

### 3.1 Descriptive statistics

Table 2 shows the means and standard deviations for the Internal-SOS-AMP dimension scores for each sexual orientation group. For the moral and normal dimensions, the heterosexual couple images elicited the highest scores, followed by queer women couple images, and then queer men couple images. Alternatively, for the appealing and pleasant dimensions, queer women couple images elicited the highest scores, followed by queer men couple images, and then heterosexual couple images.

**Table 2:** Descriptive statistics for the Internal-SOS-AMP dimension scores.

Image group and response dimension	Mean (*SD*)	
	*Wave 1* (*N* = 500)	*Wave 2* (*N* = 358)
Heterosexual couples		
Moral/immoral	0.707 (0.259)	0.695 (0.263)
Normal/abnormal	0.688 (0.254)	0.669 (0.269)
Appealing/unappealing	0.649 (0.267)	0.629 (0.277)
Pleasant/unpleasant	0.693 (0.255)	0.646 (0.271)
Same-sex female couples		
Moral/immoral	0.679 (0.290)	0.653 (0.309)
Normal/abnormal	0.667 (0.285)	0.650 (0.300)
Appealing/unappealing	0.714 (0.252)	0.677 (0.279)
Pleasant/unpleasant	0.744 (0.231)	0.719 (0.255)
Same-sex male couples		
Moral/immoral	0.648 (0.312)	0.630 (0.317)
Normal/abnormal	0.631 (0.308)	0.628 (0.313)
Appealing/unappealing	0.671 (0.281)	0.661 (0.287)
Pleasant/unpleasant	0.725 (0.252)	0.675 (0.283)

Positive responses (i.e., moral, normal, appealing, pleasant) were coded as 1, and negative responses (i.e., immoral, abnormal, unappealing, unpleasant) were coded as 0.

### 3.2 Confirmatory factor analysis

Initial CFA results showed that neither model met any of the pre-established goodness-of-fit criteria (i.e., CFI, TLI, RMSEA, and SRMR). Therefore, theoretically and statistically justifiable modifications were considered (Hu and Bentler, 1999). After examining model modification indices, four correlated error terms were allowed between four pairs of indicators that measured the same conceptual dimensions of internalized stigma (e.g., *immorality* of queer men and *immorality* of queer women) as seen in Figure 2. Allowing correlated error terms is an acceptable strategy when indicators use similar wording (Brown, 2015), which was the case with this measure using the same response option terms (e.g., “immoral” or “moral”) to capture immorality of queer men and immorality of queer women (in this example) across the two factors. Uncorrelated error terms imply that all measurement error is random; however, there may have been measurement error patterns related to response options wording. Table 3 shows the chi-square values and degrees of freedom for each model and the fit indices for the modified models based on the maximum likelihood estimation. Factor Model 1 (the one-factor model of implicit internalized queer stigma) met only one of the pre-stated model fit criteria (SRMR <0.08), whereas Factor Model 2 (the two-factor model of implicit internalized queer stigma based on two gender stimuli groups) met two of the criteria (CFI ≥ 0.95 and SRMR <0.08). Several structural equation modeling scholars have advocated for fit indices to be applied flexibly, recognizing that goodness-of-fit exists on a continuum (e.g., Niemand and Mai, 2018). Thus, we note that the TLI value of 0.94 for Factor Model 2 was very close to the cutoff value. Furthermore, although the RMSEA of 0.117 for Factor Model 2 was outside the cutoff value (RMSEA ≤0.06), a RMSEA of 0.1 could still indicate minimally adequate fit (MacCallum et al., 1996). Altogether, Factor Model 2 had a superior fit and is recommended to be retained.

**Table 3:** Goodness-of-fit statistics for the Internal-SOS-AMP Models (*N* = 500).

Model	*χ* ^2^	*df*	CFI	TLI	RMSEA (90% CI)	SRMR
Factor model 1	279.32*	16	0.92	0.86	0.181 (0.163–0.200)	0.068
Factor model 2	118.36*	15	0.97	0.94	0.117 (0.098–0.138)	0.062

CFI, comparative fit index; TLI, Tucker-Lewis index; RMSEA, root mean square error of approximation; SRMR, standardized root mean square residual. *p < 0.05.

### 3.3 Correlational results

#### 3.3.1 Correlations between Internal-SOS-AMP scores

Table 4 shows the means, standard deviations, and inter-correlations for the Internal-SOS-AMP composite scores. The Internal-SOS-AMP mean scores are close to 0.0, and the medians were all 0.0. Composite scores of 0 indicate neutral attitudes or no implicit internalized stigma. Correlations among the eight scores ranged from 0.392 to 0.853. Correlations were higher between scores measuring similar dimensions (e.g., queer women moral/immoral and queer men moral/immoral). Correlations were slightly higher between scores in the same gender stimuli stigma group (e.g., queer women stigma) than between gender stimuli stigma score correlations.

**Table 4:** Means, standard deviations, and intercorrelations for Internal-SOS-AMP scores and criterion validity variables (*N* = 500).

Variable	*M* (*SD*)	1	2	3	4	5	6	7	8	9
1. Implicit internalized stigma toward queer men (moral/immoral)	0.056 (0.38)	–								
2. Implicit internalized stigma toward queer men (normal/abnormal)	0.058 (0.38)	0.755*	–							
3. Implicit internalized stigma toward queer men (appealing/unappealing)	−0.022 (0.35)	0.532*	0.520*	–						
4. Implicit internalized stigma toward queer men (pleasant/unpleasant)	−0.033 (0.30)	0.495*	0.493*	0.612*	–					
5. Implicit internalized stigma toward queer women (moral/immoral)	0.025 (0.36)	0.853*	0.672*	0.456*	0.419*	–				
6. Implicit internalized stigma toward queer women (normal/abnormal)	0.022 (0.36)	0.666*	0.841*	0.452*	0.411*	0.693*	–			
7. Implicit internalized stigma toward queer women (appealing/unappealing)	−0.065 (0.33)	0.454*	0.437*	0.783*	0.518*	0.468*	0.478*	–		
8. Implicit internalized stigma toward queer women (pleasant/unpleasant)	−0.051 (0.27)	0.394*	0.401*	0.500*	0.734*	0.409*	0.392*	0.559*	–	
9. Explicit internalized queer stigma	1.893 (1.06)	0.097*	0.049	0.092*	0.139*	0.136*	0.092*	0.125*	0.157*	–
10. Affirmation of queer identity	5.513 (1.07)	−0.131*	−0.068	−0.194*	−0.114*	−0.127*	−0.065	−0.187*	−0.115*	−0.471*

^*^p < 0.05.

#### 3.3.2 Correlations between Internal-SOS-AMP scores and criterion validity variables

Table 4 also shows correlations between Internal-SOS-AMP scores and the criterion validity variables, which included explicit internalized queer stigma and affirmation of queer identity. Positive correlations were found between Internal-SOS-AMP scores and explicit internalized queer stigma scores; seven out of eight of these correlations were statistically significant, ranging from 0.092 to 0.157. The correlation between implicit internalized stigma toward queer men (normal/abnormal dimension) and explicit internalized queer stigma was in the expected direction but not statistically significant (*r* = 0.049). Correlations between Internal-SOS-AMP scores and affirmation of queer identity scores were all inverse; six out of eight of these correlations were statistically significant, ranging from −0.114 to −0.194. The correlations between implicit internalized stigma toward queer men and women (normal/abnormal dimensions) and affirmation of queer identity were inverse but not statistically significant (*r* = −0.068 and − 0.065, respectively).

### 3.4 Reliability analyses

Table 5 shows the internal consistency reliabilities for the Internal-SOS-AMP composite scores, which ranged from Cronbach’s *α* = 0.74 to 0.85. Table 5 also shows the ICCs for test–retest reliability between Internal-SOS-AMP scores, which ranged from 0.459 to 0.668. ICCs were higher for the moral/immoral and normal/abnormal dimensions than the appealing/unappealing and pleasant/unpleasant dimensions.

**Table 5:** Test–retest reliability and Internal consistency reliability for Internal-SOS-AMP scores.

Dimension	ICC (95% CI)	Cronbach’s alpha	
		Wave 1	Wave 2
Implicit internalized stigma toward queer women		0.80	0.87
Moral/immoral	0.668* (0.591, 0.730)		
Normal/abnormal	0.659* (0.580, 0.723)		
Appealing/unappealing	0.550* (0.446, 0.634)		
Pleasant/unpleasant	0.459* (0.334, 0.561)		
Implicit internalized stigma toward queer men		0.84	0.88
Moral/immoral	0.659* (0.580, 0.723)		
Normal/abnormal	0.626* (0.540, 0.697)		
Appealing/unappealing	0.566* (0.466, 0.648)		
Pleasant/unpleasant	0.529* (0.420, 0.617)		

ICC, Intraclass correlation coefficient. *p < 0.05.

## 4 Discussion

The aims of this study were to create a measure of implicit internalized stigma among queer people and examine its psychometric properties. We will discuss the psychometric evidence of the Internal-SOS-AMP in the order of the research questions and presentation of the results. Overall, psychometric findings show strong evidence of content validity, convergent validity, divergent validity, and reliability of the Internal-SOS-AMP.

### 4.1 Dimensions of implicit Internalized stigma in the Internal-SOS-AMP

The generation and selection of response options to capture the primary dimensions of implicit internalized queer stigma involved a thorough review of the empirical and theoretical/conceptual literature on the construct, a review of seven extant measures of internalized stigma, and interviews with experts on sexual minority stigma. Although many dimensions of stigma were identified, for the Internal-SOS-AMP, we selected primary dimensions of internalized stigma (i.e., abnormality/deviance, immorality, and negative affect). Only three of the extant measures reviewed [i.e., Internalized Homophobia Scale (Ross and Rosser, 1996); Internalized Homophobia Scale (Wagner et al., 1994; Wagner et al., 1996); Lesbian Internalized Homophobia Scale (Szymanski and Chung, 2001)] included the same range of conceptual dimensions of internalized stigma as the Internal-SOS-AMP; however, these three measures focused on explicit internalized stigma. The Sexuality IAT measures only general attributes (e.g., *wonderful*, *terrible*). Therefore, at this time, the Internal-SOS-AMP is the only implicit measure to capture multiple primary dimensions of internalized queer stigma.

### 4.2 Factor structure of the Internal-SOS-AMP

CFA findings support the factor structure of the Internal-SOS-AMP. Model fit results indicate that the two-factor model structure (i.e., implicit internalized stigma as two constructs based on two gender stimuli groups: stigma regarding queer women and stigma regarding queer men) fits the data sufficiently well and demonstrated better fit than the one-factor model (i.e., a singular construct of implicit internalized queer stigma). This suggests that there are meaningful differences in implicit internalized stigma based on the genders of queer people in terms of attitude objects. Therefore, queer individuals may have differing levels of implicit internalized stigma regarding queer men versus queer women. This may be due to differences in attitudinal dimensions based on gender. For example, there may be strong attitudes of abnormality/deviance regarding queer men due to historical psychopathologizing and criminalizing homosexuality among men and cis-heterodominant norms of masculinity (Bernstein, 2004; Drescher, 2015; Harris and Mahalik, 2023), whereas homosexuality among women may be viewed as more tolerable in the context of a hetero-patriarchal society (Herek, 2002). Support for the two-factor model structure may also stem from participants’ own membership with a gender identity. For example, a person who identifies as a cisgender woman may hold different attitudes of queer women versus queer men due to their shared membership with an attitude object group, which is supported in the empirical literature (e.g., Devos and Banaji, 2003). Nonetheless, results also showed that stigma regarding queer women and stigma regarding queer men are highly related as evidenced by the strong inter-factor correlation.

Also related to factor structure, all of the standardized factor loadings for the items on their respective factors are high (i.e., 0.56 to 0.85) in the final model; this indicates that the data support the factor structure of the Internal-SOS-AMP. Correlations in scores between indicators of the Internal-SOS-AMP were moderate to strong (i.e., 0.39 to 0.85). These substantial associations between indicators, yet lack of very strong correlations (i.e., *r* ≥ 0.9), suggest that the indicators are measuring various facets of a construct without overlap (*cf.*, Swerdlik and Cohen, 2005).

### 4.3 Convergent and divergent validity of the Internal-SOS-AMP

The results show positive associations (*r* = 0.05 to 0.16) between Internal-SOS-AMP scores and explicit internalized queer stigma scores, which are small effect sizes (Cohen, 1988). These findings are consistent with extant evidence on the relationship between implicit attitudes and explicit attitudes, which generally shows small positive correlations between the constructs (e.g., Cameron et al., 2012; Hofmann et al., 2005; Nosek and Smyth, 2007). These findings underscore that implicit and explicit internalized stigma are distinct yet weakly related constructs and provide evidence of convergent validity for the Internal-SOS-AMP.

Results also show inverse associations (*r* = −0.07 to −0.19) between Internal-SOS-AMP scores and affirmation of queer identity, which are small to moderately small effect sizes (Cohen, 1988). These associations were theoretically expected because numerous studies show small to large inverse associations between internalized stigma and affirmation of identity among queer people (e.g., Cramer et al., 2018; Dyar and London, 2018; Feinstein et al., 2021; Paul et al., 2014; Sarno et al., 2020; Toomey et al., 2016). Therefore, these results provide evidence of divergent validity for the Internal-SOS-AMP. Two of the correlations between implicit internalized stigma toward queer men and women (normal/abnormal dimensions) and affirmation of queer identity were inverse but not statistically significant (*p* = 0.15 and 0.13, respectively). The lack of statistical significance may indicate that abnormality attitudes regarding same-gender couples have become less connected with positive views of one’s queer identity. This may be due to advancements in civil rights for queer people in recent decades, including the *Obergefell v. Hodges* decision by the U.S. Supreme Court that legalized same-sex marriage, and overall positive shifts in attitudes about same-gender relationships (Gallup, 2023).

### 4.4 Reliability of the Internal-SOS-AMP

Internal consistency reliability results for the Internal-SOS-AMP (Cronbach’s *α* = 0.74 to 0.85) show good reliability (DeVellis and Thorpe, 2022). These findings contrast with results from the other measure of implicit internalized queer stigma (i.e., the Sexuality IAT), in which internal consistency reliability was unacceptable (i.e., Cronbach’s *α* < 0.60; DeVellis and Thorpe, 2022) or unreported in studies (Anselmi et al., 2015; Bankoff et al., 2015; Chen and Chang, 2020; Dhabhar and Deshmukh, 2021; Fleming and Burns, 2016; Hatzenbuehler et al., 2009; Jellison et al., 2004; Jones and Devos, 2014; Millar et al., 2016). High internal consistency reliability values suggest that the indicators used to capture a construct are highly interrelated (Kline, 2005).

Regarding test–retest reliability, the ICCs for moral/immoral and normal/abnormal dimensions (ICC = 0.63 to 0.67) were good, and the ICCs for appealing/unappealing and pleasant/unpleasant dimensions (ICC = 0.46 to 0.57) were fair (Cicchetti and Sparrow, 1981; Fleiss, 1999). These findings suggest that there is adequate consistency in scores between administrations of the Internal-SOS-AMP. Many implicit measures show moderate instability in scores over time (e.g., Cunningham et al., 2001; Devine et al., 2012; Gawronski et al., 2017), and implicit measures may have poorer test–retest reliability than explicit measures (Gawronski et al., 2017). Implicit measures may be more susceptible to the influence of contextual factors present during measure administration than explicit measures, leading to somewhat low test–retest reliabilities. More research is needed to identify these contextual factors.

### 4.5 Limitations

This study has several limitations. Although the sample is a moderately large, national sample, it is not nationally representative. On average, participants were slightly younger and more middle class than the general population. White participants were slightly over-represented and Hispanic/Latine participants were under-represented. Therefore, caution should be taken when generalizing results. A limitation of the Internal-SOS-AMP is that it does not capture the full range of implicit internalized stigma that may be present in the queer community, which is a heterogeneous community. For example, there may be negative attitudes toward bisexual or pansexual people who are in relationships with other-gender partners and may be perceived to be in a heterosexual relationship; such attitudes are not captured with the Internal-SOS-AMP. In addition, the lack of images of older queer couples may have failed to capture stigma regarding older queer people, which may result from intersections of heterosexism and ageism. The measure is also limited in the conceptualization of internalized stigma, being primarily informed by U.S. literature and data; this construct may manifest differently in various cultures and nations across the globe. Another study limitation was the RMSEA of the retained model was not ideal. A constraint of the Internal-SOS-AMP is that the prime images focused on those who appear to be cisgender men and women same-gender couples. An absence of gender diverse people (e.g., transgender, genderqueer, and non-binary people) in images of couples may reinforce cisnormativity in relationships. Gender diverse people are a distinct group within the broader queer community; therefore, there are likely distinct implicit internalized attitudes about gender diverse people, which should be measured with a corresponding implicit measure (e.g., Kanamori et al., 2020).

### 4.6 Future research

Though this study provides strong evidence of validity and reliability of the Internal-SOS-AMP, additional validation research is needed to evaluate its measurement properties in different ways with different samples. Future work could investigate the measurement variance/invariance of the Internal-SOS-AMP across subgroups of the queer community, including by gender, sexual orientation, age/developmental period, and race/ethnicity. Research could also disaggregate Internal-SOS-AMP scores by couple race/ethnicity and examine potential differences in implicit attitudes of White versus couples of color. Future work should examine the predictive validity of the Internal-SOS-AMP. Evidence syntheses show that internalized stigma measured explicitly is significantly associated with psychological problems, including depression, suicidal ideation and behavior, anxiety symptoms, and non-suicidal self-injurious behavior, among queer people in cross-sectional and longitudinal studies (Hall, 2018; Newcomb and Mustanski, 2010; Szymanski et al., 2008b; Williams et al., 2023). Researchers should examine the extent that Internal-SOS-AMP scores predict mental and behavioral health problems. Another area for future research is examining the practice-related validity (Bowen, 2008) of the Internal-SOS-AMP. Internalized stigma is a pernicious psychological problem that is difficult to assess with traditional means for research or clinical purposes. Given the established relations between internalized stigma and mental health inequities among queer people, mental health practitioners working with queer clients/patients may wish to use the Internal-SOS-AMP to detect implicit internalized stigma for assessment and treatment purposes. Data on the practicality and utility of the measure for these means can be collected and examined. Indeed, measures with empirical support for validity and reliability are necessary for the development of epidemiological research and intervention efforts as well as accurate clinical assessment about mental health needs. Measures can play a role in eliminating the mental health inequities within the queer community.

## Data Availability

The raw data supporting the conclusions of this article will be made available by the authors, without undue reservation.
